# Mitigating Effect of 1-Palmitoyl-2-Linoleoyl-3-Acetyl-Rac-Glycerol (PLAG) on a Murine Model of 5-Fluorouracil-Induced Hematological Toxicity

**DOI:** 10.3390/cancers11111811

**Published:** 2019-11-18

**Authors:** Jinseon Jeong, Yong-Jae Kim, Do Young Lee, Ki-Young Sohn, Sun Young Yoon, Jae Wha Kim

**Affiliations:** 1 Division of Systems Biology and Bioengineering, Korea Research Institute of Bioscience and Biotechnology, Daejeon 34141, Korea; 2 Department of Functional Genomics, University of Science & Technology, Daejeon 34113, Korea; 3 Division of Global New Drug Development, ENZYCHEM Lifesciences, Jecheon 27159, Korea

**Keywords:** PLAG, chemotherapy-induced hematological toxicity, neutropenia, monocytopenia, thrombocytopenia, thrombocytosis, 5-Fluorouracil

## Abstract

5-Fluorouracil (5-FU) is an antimetabolite chemotherapy widely used for the treatment of various cancers. However, many cancer patients experience hematological side effects following 5-FU treatment. Here, we investigated the protective effects of 1-palmitoyl-2-linoleoyl-3-acetyl-rac-glycerol (PLAG) as a mitigator against 5-FU-induced hematologic toxicity, including neutropenia, monocytopenia, thrombocytopenia, and thrombocytosis, in Balb/c mice injected with 5-FU (100 mg/kg, i.p.). Administration of PLAG significantly and dose-dependently reduced the duration of neutropenia and improved the nadirs of absolute neutrophil counts (ANCs). Moreover, while the ANCs of all mice in the control fell to the severely neutropenic range, none of the mice in the PLAG 200 and 400 mg/kg-treated groups experienced severe neutropenia. Administration of PLAG significantly delayed the mean first day of monocytopenia and reduced the duration of monocytopenia. PLAG also effectively reduced extreme changes in platelet counts induced by 5-FU treatment, thus preventing 5-FU-induced thrombocytopenia and thrombocytosis. PLAG significantly decreased plasma levels of the chemokine (C–X–C motif) ligand 1 (CXCL1), CXCL2, interleukin (IL)-6, and C-reactive protein (CRP), which were elevated consistently with the occurrence time of neutropenia, monocytopenia, and thrombocytopenia. When compared with olive oil and palmitic linoleic hydroxyl glycerol (PLH), only PLAG effectively mitigated 5-FU-induced hematological toxicity, indicating that it has a distinctive mechanism of action. In conclusion, PLAG may have therapeutic potential as a mitigator for 5-FU-induced neutropenia and other hematological disorders.

## 1. Introduction

5-Fluorouracil (5-FU) is a pyrimidine analog of antimetabolite drug used for the treatment of various solid tumors including colorectal cancer, head and neck cancer, and breast cancer [1]. Although 5-FU has been widely used as a first line of chemotherapy to improve tumor elimination and survival [2,3], many patients who received this drug have experienced a range of side effects [4,5,6]. Among these, hematological toxicity occurs very frequently in cancer patients treated with chemotherapy, and it is a major cause of treatment discontinuation [7]. The cytotoxic chemotherapy decreases white blood cells (neutropenia and leukopenia), platelets (thrombocytopenia), and red blood cells (anemia), which may cause detrimental effects to patients [8,9,10]. These complications result in dose reductions or delay of the scheduled chemotherapy cycles, which can compromise treatment efficacy and even mortality [11]. Therefore, it is important to prevent the development of chemotherapy-induced hematological toxicity in cancer patients.

Chemotherapy-induced neutropenia is a major dose-limiting toxicity that predisposes cancer patients to infectious diseases with bacteria, such as *E. coil* and *Pseudomonas*, and fungi, such as *Candida* and *Aspergillus* [12]. Current treatment options for neutropenic cancer patients are the use of hematopoietic growth factors including the recombinant human granulocyte-colony stimulating factor (rhG-CSF) [13,14]. The use of the cytokine has greatly advanced the management of chemotherapy-induced neutropenia, however, adverse side effects associated with rhG-CSF have been reported [15,16,17]. Furthermore, the adjuvant use of G-CSF should be strictly prescribed because it was reported to modulate the progression of certain non-myeloid tumors [18]. According to Morris and his colleagues, G-CSF receptor (G-CSFR) are highly expressed in human gastrointestinal tumors, and G-CSF is highly produced by stromal myofibroblasts and cancer cells. Thus, the activation of the G-CSF/R axis increases the proliferation and migration of cancer cells by expressing stem-like markers [19]. Therefore, the use of rhG-CSF for the mitigation of chemotherapy-induced neutropenia may be accompanied by unwanted side effects.

Chemotherapy-induced thrombocytopenia is another serious complication that results in dose reduction or discontinuation of chemotherapy [20]. In published reports, about 3–4% of chemotherapy-treated cancer patients experience grade IV thrombocytopenia (platelet counts < 250,000/μL) that requires platelet transfusions [9]. The current treatment option for chemotherapy-induced thrombocytopenia is to use thrombopoietic agents, such as eltrombopag and romiplostim [21,22]. These agents must be delivered prophylactically, in order to prevent the development of chemotherapy-induced thrombocytopenia, because the time needed for the agents to differentiate hematopoietic progenitors to megakaryocytes is about 10–14 days [23]. Recent studies demonstrated that the thrombopoietin receptor agonists reduce the degree and duration of chemotherapy-induced thrombocytopenia, thus allowing the scheduled chemotherapy dose and cycles [24]. However, the long-term effects of these agents on the survival and well-being of cancer patients have not been established.

1-palmitoyl-2-linoleoyl-3-acetyl-rac-glycerol (PLAG), a monoacetyldiglyceride, is a synthetic lipid molecule produced by reacting glycerol, palmitic acid, and linoleic acid [25]. Previously, PLAG has been demonstrated in various inflammatory diseases, such as hepatitis, asthma, rheumatoid arthritis, and oral mucositis [26,27,28,29]. Recently, we showed that administration of PLAG effectively mitigates hematopoietic acute radiation syndrome of mice following a lethal dose of gamma-ray irradiation [30]. Besides, PLAG effectively reduced the incidence of gemcitabine-induced neutropenia by attenuating IL-8-induced neutrophil extravasation in clinical and nonclinical settings [31,32]. Here, we investigated whether PLAG attenuates chemotherapy-induced hematological toxicity in another antimetabolite drug, 5-FU-based chemotherapy. Therefore, the purpose of this study is to present the effect of PLAG in the attenuation of chemotherapy-induced hematological toxicity by analyzing the kinetics of neutrophils, monocytes, and platelets in the peripheral blood over a 15-day observation period after intraperitoneal injection of 5-FU. We also suggest that there is a close association between the occurrence of 5-FU-induced hematological toxicity and elevated plasma levels of pro-inflammatory cytokine/chemokines.

## 2. Results

### 2.1. The Administration of PLAG Attenuates 5-FU-Induced Neutropenia

**Figure 1:**
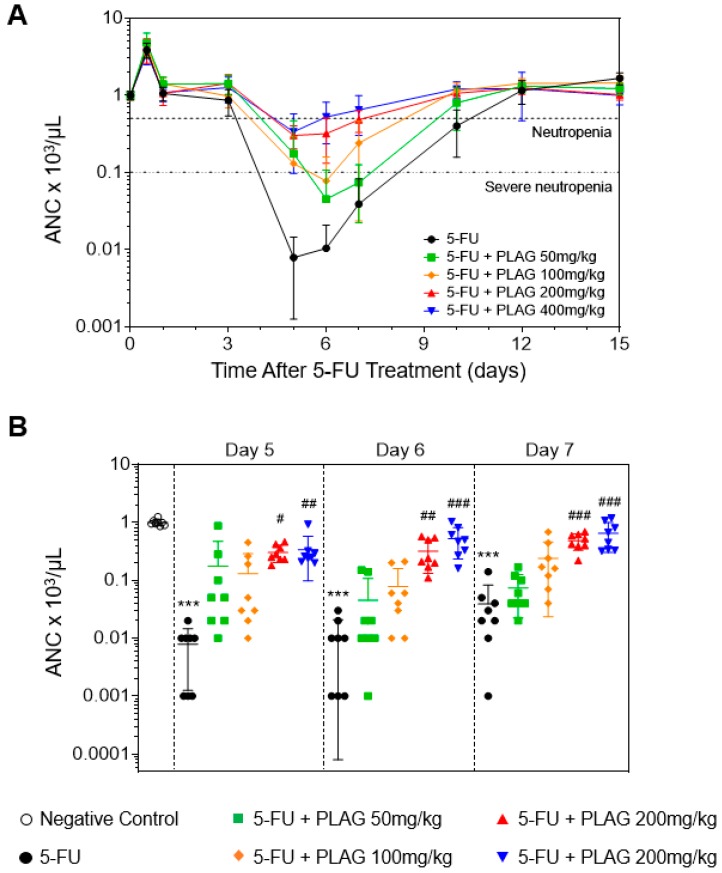
1-palmitoyl-2-linoleoyl-3-acetyl-rac-glycerol (PLAG) mitigates 5-Fluorouracil (5-FU)-induced neutropenia. Mice (*n* = 8 males per group) were intraperitoneally injected to 100 mg/kg of 5-FU immediately followed by oral administration of 50, 100, 200, and 400mg/kg of PLAG and continuing daily to day 15. (**A**) Effect of PLAG administration on the kinetics of the absolute neutrophil counts (ANCs) after 5-FU injection for 15 days. (**B**) Individual ANC data are presented as dots on days 5, 6, and 7. * indicates negative control vs. 5-FU and # indicates 5-FU vs. 5-FU + PLAG-treated groups. */# *p* < 0.05, **/## *p* < 0.01, ***/### *p* < 0.001. The complete blood counts (CBCs) data are representative of five independent experiments with eight mice per group.

A single injection of 5-FU 100 mg/kg reduced the absolute neutrophil counts (ANCs) in the control from pre-injection values to <500 cells/μL by 4.7 ± 0.2 days, and PLAG did not significantly delay the onset time of neutropenia (Figure 1A and Table 1). However, the administration of PLAG in 5-FU-injected mice resulted in significant and dose-dependent reduction in the duration of neutropenia (Table 1). As shown in Figure 1B, the control group experienced severe neutropenia (ANC < 100 cells/μL) on days 5, 6, and 7, and administration of PLAG significantly attenuated 5-FU-induced depletion of ANC in a dose-dependent manner. Moreover, the ANC of all mice in the control fell to severely neutropenic range, while none of the mice in the PLAG 200 and 400 mg/kg-treated groups experienced severe neutropenia (Figure 1A and Table 2). Administration of PLAG 50 and 100 mg/kg also reduced the duration of severe neutropenia from 5.1 ± 0.5 days to 3.2 ± 0.6 and 2.1 ± 0.6 days, respectively (Table 2). PLAG administration significantly and dose-dependently increased the mean ANC nadir (Table 3). The time of recovery to an ANC ≥500 was significantly and dose-dependently reduced in PLAG-treated groups (Table 3). From these observations, PLAG is very effective in preventing 5-FU-induced neutropenia.

### 2.2. The Administration of PLAG Attenuates 5-FU-Induced Monocytopenia

**Figure 2:**
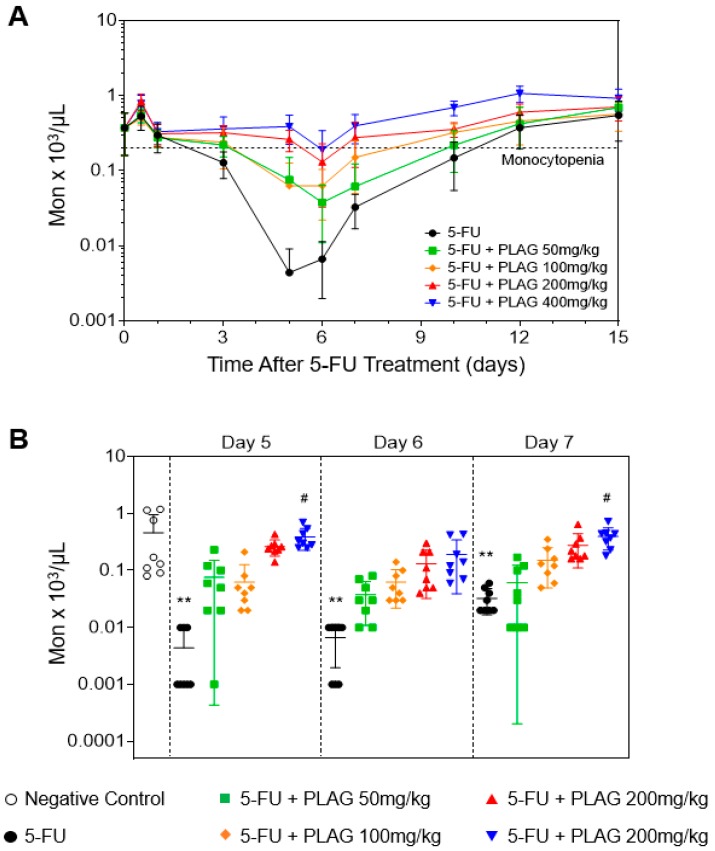
PLAG mitigates 5-FU-induced monocytopenia. Mice (*n* = 8 males per group) were intraperitoneally injected to 100 mg/kg of 5-FU immediately followed by oral administration of 50, 100, 200, and 400 mg/kg of PLAG, continuing daily until day 15. (**A**) Effect of PLAG administration on the kinetics of peripheral monocytes after 5-FU injection for 15 days. (**B**) The peripheral monocyte counts are presented as dots on days 5, 6, and 7. The complete blood counts (CBCs) data are representative of five independent experiments with eight mice per group. * indicates negative control vs. 5-FU, and # indicates 5-FU vs. 5-FU + PLAG-treated groups. */# *p* < 0.05, **/## *p* < 0.01, ***/### *p* < 0.001. ns., not significant.

As monocytes and neutrophils are differentiated from a common myeloid progenitor, they are closely associated with one another [33]. It was reported that monocyte nadir can be used as a predictive factor for neutrophil nadir during lung cancer chemotherapy, and that early onset of monocytopenia after chemotherapy is a risk factor for neutropenia [34]. Therefore, we investigated the effect of PLAG on the kinetics of peripheral monocytes in 5-FU-injected mice. Monocytopenia is defined as a peripheral monocyte count of less than 150 cells/μL [35]. The onset time of monocytopenia in 5-FU-injected mice was 3.7 ± 0.4 days, which occurred about one day earlier than that of neutropenia. The administration of PLAG in 5-FU-injected mice significantly and dose-dependently delayed the onset time of monocytopenia (Figure 2A, Table 1). In addition, PLAG significantly reduced the duration of monocytopenia in a dose-dependent manner (Table 1). As shown in Figure 2B, administration of PLAG significantly attenuated 5-FU-induced depletion of peripheral monocyte counts in a dose-dependent manner on days 5, 6, and 7. Moreover, PLAG administration significantly and dose-dependently increased the mean monocyte nadir after 5-FU injection (Table 3). The time of recovery to the peripheral monocyte counts ≥150 cells/μL was significantly and dose-dependently reduced in PLAG-treated groups (Table 3). From these observations, PLAG is very effective in preventing 5-FU-induced monocytopenia followed by neutropenia by attenuating 5-FU-induced reduction in the peripheral monocyte counts. 

### 2.3. The Administration of PLAG Attenuates 5-FU-Induced Aberrant Changes in Platelet Counts

**Figure 3:**
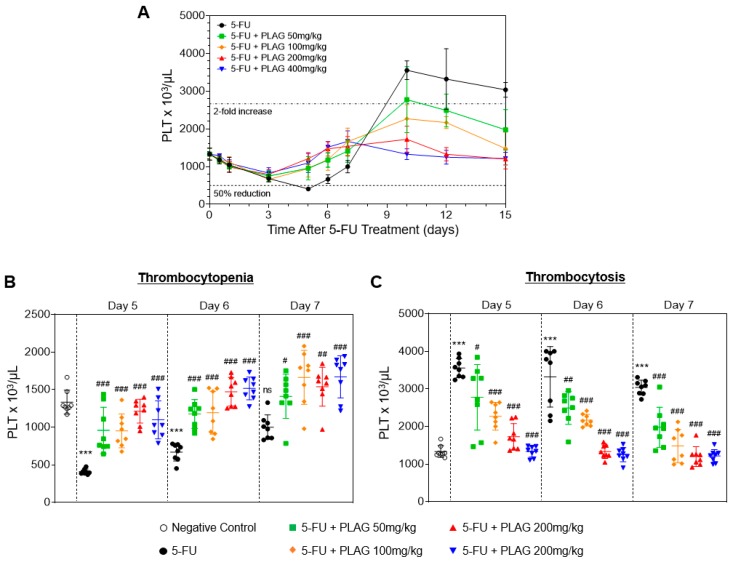
PLAG prevents 5-FU-induced thrombocytopenia and thrombocytosis. Mice (*n* = 8 males per group) were intraperitoneally injected to 100 mg/kg of 5-FU immediately followed by oral administration of 50, 100, 200, and 400 mg/kg of PLAG, continuing daily until day 15. (**A**) Effect of PLAG administration on the kinetics of peripheral platelets after 5-FU injection for 15 days. The peripheral platelet counts are presented as dots (**B**) on days 5, 6, and 7 and (**C**) on days 10, 12, and 15. The complete blood counts (CBCs) data are representative of five independent experiments with eight mice per group. * indicates negative control vs. 5-FU and # indicates 5-FU vs. 5-FU + PLAG-treated groups. */# *p* < 0.05, **/## *p* < 0.01, ***/### *p* < 0.001. ns., not significant.

It has long been demonstrated that 5-FU induces a moderate reduction of platelet counts (thrombocytopenia), followed by a profound and prolonged increase of platelet counts (thrombocytosis) [36]. Therefore, we investigated the kinetics of peripheral platelets and the effect of PLAG on the aberrant changes in platelet counts in 5-FU-injected mice. In this study, we defined thrombocytopenia as a 50% or greater reduction in platelet count from baseline (666 × 10^3^ cells/μL), and a twofold or greater increase of platelet count from baseline (2664 × 10^3^ cells/μL) for thrombocytosis. A single injection of 100 mg/kg of 5-FU induced the moderate thrombocytopenia from three to six days, followed by a more pronounced and prolonged rebound thrombocytosis (Figure 3). PLAG did not significantly influence the onset time of thrombocytopenia (Table 1). However, the platelet counts of all mice in the control fell to the thrombocytopenic range, while the number of mice in PLAG-treated groups remarkably decreased to 3, 4, 1, and 1 out of 8 mice, respectively. PLAG administration significantly and dose-dependently increased the mean nadir of platelet counts after 5-FU injection (Table 3). Moreover, the time of recovery to platelet counts ≥1000 × 10^3^ cells/μL was significantly and dose-dependently reduced in PLAG-treated groups (Table 3). As shown in Figure 3B, administration of PLAG significantly attenuated 5-FU-induced depletion of platelet counts in a dose-dependent manner on days 5, 6, and 7. After the moderate thrombocytopenia, 5-FU induced a remarked increase of platelet counts, which was sustained until the end of the observation (Figure 3A). Administration of PLAG significantly and dose-dependently reduced 5-FU-induced increase of platelet counts on days 10, 12, and 15 (Figure 3C). In conclusion, PLAG is very effective in regulating the extreme change in platelet counts, thus preventing 5-FU-induced thrombocytopenia and thrombocytosis.

### 2.4. Correlation Between Hematology and Pro-Inflammatory Cytokine/Chemokines 

**Figure 4:**
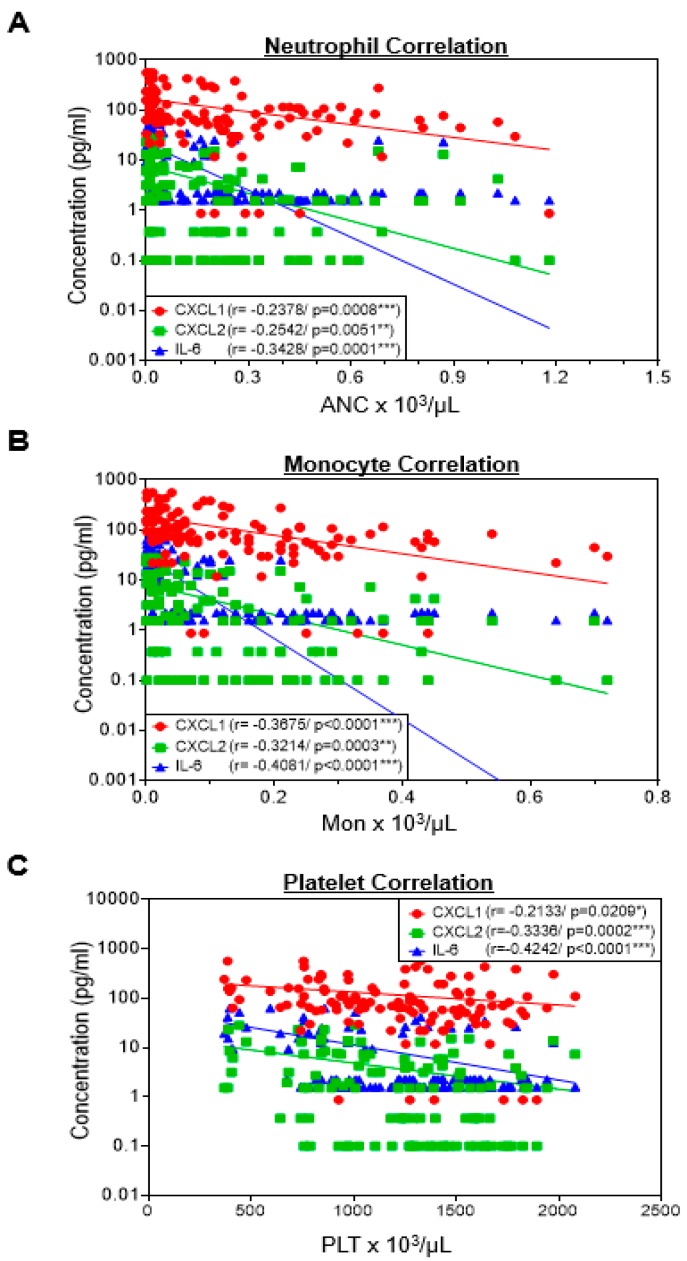
Correlation between hematology and pro-inflammatory cytokine/chemokines in 5-FU-treated mice. The blood samples were harvested from mice (*n* = 40 males per day) on days 5, 6, and 7 after 5-FU (100 mg/kg) treatment. Correlation plot between (**A**) ANCs, (**B**) monocytes, or (**C**) platelets and pro-inflammatory cytokine/chemokines the chemokine (C–X–C motif) ligand 1 (CXCL1), CXCL2, and interleukin (IL)-6 (Pearson’s correlations).

There have been many reports that 5-FU-based chemotherapy induces various inflammatory diseases, such as oral mucositis, hepatic steatosis, and intestinal mucositis [37,38,39]. However, the association between the occurrence of 5-FU-induced hematological toxicity and inflammation remains elusive. Therefore, we analyzed a correlation between hematology data and plasma levels of pro-inflammatory cytokine/chemokines including the chemokine (C–X–C motif) ligand 1 (CXCL1), CXCL2, and interleukin-6 (IL-6), which are directly associated with the migration and activation of innate immune cells, on days of 5, 6, and 7 after 5-FU treatment. As shown in Figure 4A, decreasing number of ANCs showed a trend of a negative correlation with elevated levels of CXCL1 (r = −0.2378, *p* = 0.0008), CXCL2 (r = −0.2542, *p* = 0.0051), and IL-6 (r = −0.3428, *p* = 0.0001). The decreasing number of peripheral monocyte counts showed a more significant negative correlation with elevated levels of CXCL1 (r = −0.3675, *p* < 0.0001), CXCL2 (r = 0.3214, *p* = 0.0003), and IL-6 (r = 0.4081, *p* < 0.0001) (Figure 4B). Moreover, a decreasing number of peripheral platelet counts suggests a negative correlation with elevated levels of CXCL1 (r = −0.2133, *p* = 0.0209), CXCL2 (r = −0.3336, *p* = 0.0002), and IL-6 (r = −0.4242, *p* < 0.0001) (Figure 4C). These observations indicate that the development of 5-FU-induced hematology toxicity may have a correlation with inflammatory responses. 

### 2.5. The Administration of PLAG Attenuates Blood Levels of 5-FU-Induced Pro-Inflammatory Cytokine/Chemokines and C-Reactive Protein (CRP)

**Figure 5:**
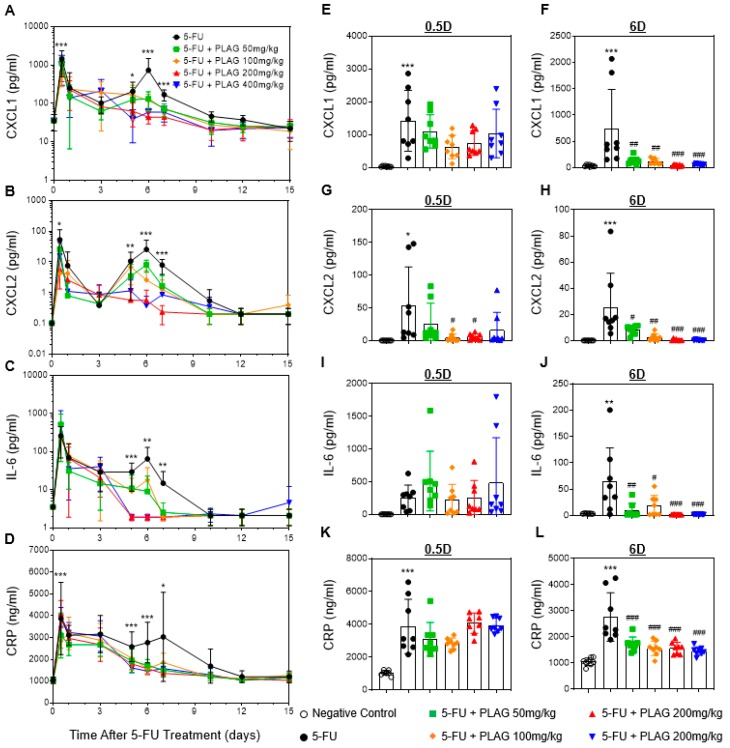
PLAG attenuates blood levels of 5-FU-induced pro-inflammatory cytokine/chemokines and C-reactive protein (CRP). Mice (*n* = 8 males per group) were intraperitoneally injected to 100 mg/kg of 5-FU immediately followed by oral administration of 50, 100, 200, and 400 mg/kg of PLAG, continuing daily until day 15. Effect of PLAG administration on the kinetics of (**A**) the chemokine (C–X–C motif) ligand 1 (CXCL1), (**B**) CXCL2, (**C**) interleukin-6 (IL-6), and (**D**) CRP in blood after 5-FU injection. Individual data of CXCL1, CXCL2, IL-6, and CRP on 0.5 day (**E**,**G**,**I**,**K**) and six days (**F**,**H**,**J**,**L**) after 5-FU injection are presented as dots. The luminex and ELISA data are representative of five independent experiments with eight mice per group. * indicates negative control vs. 5-FU and # indicates 5-FU vs. 5-FU + PLAG-treated groups. */# *p* < 0.05, **/## *p* < 0.01, ***/### *p* < 0.001. ns., not significant.

As there is a negative correlation between the occurrence of 5-FU-induced hematological toxicity and the increase of pro-inflammatory cytokine/chemokines, we next evaluated the kinetics of plasma CXCL1, CXCL2, and IL-6. 5-FU induced two significant peaks of the pro-inflammatory cytokine/chemokines. The plasma levels of CXCL1, CXCL2, and IL-6 were significantly elevated by about 40.5-, 521.6-, and 145.1-fold on day 0.5 (*p* < 0.001), and by about 20.7-, 253.03-, and 18.3-fold on day 6 (*p* < 0.001), respectively, after 5-FU injection (Figure 5A–C). The second peak of the pro-inflammatory cytokine/chemokines on day 6 after 5-FU injection was consistent with the occurrence time of neutropenia, monocytopenia, and thrombocytopenia. While PLAG did not affect the first peak of the pro-inflammatory cytokine/chemokines on day 0.5 (Figure 5E,G,I), the levels of CXCL1, CXCL2, and IL-6 were significantly and dose-dependently attenuated by PLAG on day 6 (Figure 5F,H,J). The appearance of fever in neutropenic patients, called febrile neutropenia, is a serious complication that requires immediate hospitalization [40]. Therefore, we also examined the plasma level of C-reactive protein (CRP), which is a well-known marker for febrile neutropenia [41,42]. As with the pro-inflammatory cytokine/chemokines, CRP had two significant peaks over a 15-day observation period after 5-FU treatment (Figure 5D). PLAG significantly and dose-dependently attenuated 5-FU-induced CRP production on day 6 (Figure 5L). In conclusion, PLAG effectively attenuates the production of pro-inflammatory cytokine/chemokines, which is associated with the occurrence of 5-FU-induced hematological toxicity. In addition, PLAG prevents the development of febrile neutropenia by reducing the plasma level of CRP. 

### 2.6. Comparative Analysis between Administration of PLAG and Olive Oil in 5-FU-Induced Hematological Toxicity

**Figure 6:**
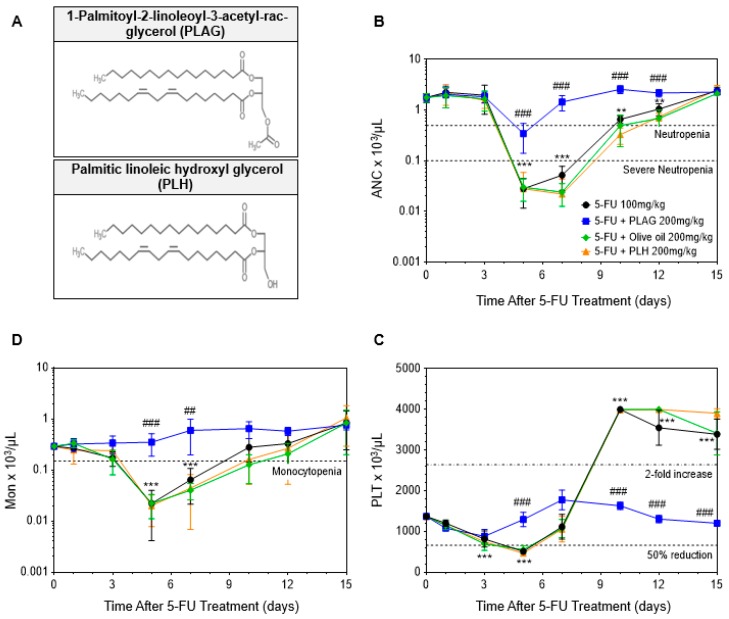
Effect of PLAG, olive oil, and PLH in 5-FU-induced hematological toxicity. Mice (*n* = 5 males per group) were intraperitoneally injected to 100 mg/kg of 5-FU, immediately followed by oral administration of 200 mg/kg of PLAG, olive oil, or PLH, continuing daily until day 15. (**A**) The chemical structure of PLAG and PLH. The effect of PLAG, olive oil, or PLH administration on the kinetics of (**B**) ANC, (**C**) monocytes, and (**D**) platelets after 5-FU injection. The CBC data are representative of three independent experiments with five mice per group. * indicates negative control vs. 5-FU and # indicates 5-FU vs. 5-FU + PLAG-treated groups. */# *p* < 0.05, **/## *p* < 0.01, ***/### *p* < 0.001. ns, not significant.

PLAG is an 1,2-acyl-3-acetylglycerol-type lipid molecule in which palmitic acid and linoleic acid are esterified to the first and second position of glycerol backbone, respectively, and the acetyl acid to the third positon (Figure 6A). As PLAG is a lipid molecule with a calorie of 883 kcal/100g, we next investigated whether the mitigating effect of PLAG resulted from the extra calories of the fatty acids by comparison with olive oil, which contains 884.1 kcal/100g. We also compared PLAG with its unacetylated form, palmitic linoleic hydroxyl glycerol (PLH), to investigate whether its mitigating effect comes from the acetyl moiety. The comparison between PLAG, olive oil, and PLH administration in 5-FU-induced hematological toxicity is presented in Table 4. PLAG was far superior to olive oil and PLH in mitigating 5-FU-induced neutropenia (Figure 6B). While PLAG significantly reduced the duration of neutropenia from 5.0 ± 0.0 to 2.0 ± 0.0 days, administration of olive oil and PLH had no significant effect in reducing the duration of neutropenia. Moreover, while none of the mice in PLAG-treated group experienced severe neutropenia, all mice in the control, olive oil-treated, and PLH-treated group experienced severe neutropenia. PLAG significantly increased the mean ANC nadir compared with the control, but olive oil and PLH did not (Table 4). As shown in Figure 6C, PLAG was also more effective than olive oil and PLH in mitigating 5-FU-induced monocytopenia. While PLAG effectively improved the duration of monocytopenia and monocyte nadir, olive oil and PLH did not. While PLAG effectively prevented the extreme change of platelet counts induced by 5-FU treatment, olive oil and PLH did not (Figure 6D). Only PLAG significantly increase the mean nadir of platelet counts when compared with the control (Table 4). As a result, the administration of olive oil and PLH had no effects in mitigating 5-FU-induced hematological toxicity, indicating that the therapeutic effect of PLAG did not attribute to the extra calories and that the acetyl moiety of PLAG may exert its therapeutic efficacy. This observation suggests that PLAG might have a distinct mechanism in attenuating 5-FU-induced hematological toxicity.

## 3. Discussion

Cytotoxic chemotherapy develops variable hematological toxicity by suppressing or impairing the hematopoietic system [43]. Neutropenia and monocytopenia increase the risk of infection by weakening the inflammatory response to early infections, allowing bacterial proliferation and invasion [44]. Also, thrombocytopenia and thrombocytosis result in increased risks of excessive bleeding and thrombosis, respectively [20,45]. However, the most immediate danger is that these hematopoietic complications eventually lead to a reduction in chemotherapy dose and frequency, compromising the patients’ long-term disease-free survival [14,43]. In this study, we demonstrated the mitigating effect of PLAG on 5-FU-induced hematological toxicity and elevation of pro-inflammatory cytokine/chemokines.

In previous studies, PLAG was both clinically and non-clinically verified to attenuate gemcitabine-induced neutropenia [31,32]. PLAG attenuated the degree of neutropenia and excessive neutrophil extravasation into the peritoneal cavity by reducing gemcitabine-generated reactive oxygen species (ROS) followed by macrophage inflammatory protein 2 (MIP-2) production [31]. In this study, we used 5-FU-based chemotherapy, which is known to induce more prolonged neutropenia [46], to verify whether PLAG attenuates the duration and severity of 5-FU-induced hematological toxicity. Preclinical toxicity studies in small animals confirmed that a single oral administration of PLAG is safe and well tolerated up to 2000 mg/kg. All the safety and toxicity data of PLAG were submitted to U.S. Food and Drug Administration (FDA) for clinical trial for chemotherapy-induced neutropenia in 2015, and it is currently under a phase II clinical trial for the management of severe chemotherapy-induced neutropenia in advanced breast cancer patients who are receiving highly toxic chemotherapy [25]. 

We verified that the mitigating effect of PLAG did not result from the extra calorie of fatty acids by comparing it with olive oil, which has a similar amount of calories per weight. Unlike PLAG, olive oil is composed of an array of lipids including triacylglycerols (~99%), mono- and diacylglycerols, and free fatty acids [47]. Fatty acids present in olive oil are mostly mono- and polyunsaturated fatty acids, such as oleic (C18:1), linoleic (C18:2), and linolenic (C18:3) acids [48]. Although these fatty acids are known to have beneficial effects on health [47,49], there was no effect on the mitigation of 5-FU-induced hematological toxicity. From these observations, we perceived that the long-chain fatty acids present in PLAG were not key elements that exert therapeutic effects. PLH is a diacylglycerol consisting of palmitic and linoleic acids covalently bonded to the first and second position of the glycerol backbone, respectively. By comparing PLAG with PLH, we confirmed that the therapeutic efficacy of PLAG might result from acetyl acids esterified to the third position of the glycerol backbone. 

In this study, we administered mice with 5-FU by intraperitoneal injection to develop 5-FU-induced hematological toxicity. 5-FU induced a rapid increase of damage-associated molecular patterns (DAMPs) including s100A8/9 and lactate dehydrogenase (LDH) as well as CXCL1, CXCL2, and IL-6 in the peritoneal fluids within 24 h (data not shown). Along with the pro-inflammatory cytokine/chemokines, a primary function of DAMPs is alerting the body about danger by stimulating the immune system [50]. Once in the extracellular environment, DAMPs activate immune cells by interacting with multiple receptors and trigger inflammation in the damaged site. The activated immune cells infiltrate the damaged sites in order to eliminate cellular debris or DAMPs and enable the damaged tissues to undergo the regeneration process [51]. Finally, the damaged sites return to homeostasis, and this process is called the resolution of inflammation [52]. However, when DAMPs in the damaged tissues are not cleared timely by phagocytic immune cells, an excessive amount of neutrophils infiltrates the site of injury, which develops into chronic inflammation and further tissue injury [53]. We observed that PLAG effectively decreased 5-FU-induced release of s100A8/9 and LDH as well as CXCL1, CXCL2, and IL-6 in the peritoneal fluid (data not shown). We assume that the putative mechanism of PLAG in 5-FU-induced hematological toxicity is to accelerate the resolution of inflammation by activating phagocytic cells to phagocytose and remove cellular debris and DAMPs produced by 5-FU treatment and by preventing further tissue damages from DAMP-induced secondary inflammatory responses and excessive migration of the immune cells. We think that such a mechanism of action of PLAG would result in the positive data on hematology and plasma cytokine/chemokine analysis in our 5-FU-induced hematological toxicity model.

Generally, it has long been considered that chemotherapy-induced hematological toxicity mainly results from myeloablative effects of cytotoxic chemotherapy [54]. Indeed, 5-FU (150 mg/kg) injection to adult Swiss mice induced an apparent reduction of bone marrow cellularity, as well as the exhaustion of hematopoietic stem cells (HSCs) on day 4 after treatment [55]. We have not yet checked whether PLAG prevents 5-FU from exerting its myeloablative effects in the bone marrow, however, on the basis of our previous data, we do not think that PLAG interrupts the cytotoxic activity of 5-FU on both primary tumors and bone marrow. In a murine model of gemcitabine-induced neutropenia, we demonstrated that PLAG ameliorates ANC decrease without interfering with the tumor-killing effect of gemcitabine [31]. Instead, we assume that PLAG accelerates the recovery of bone marrow cellularity after chemotherapy-induced myeloablation because monoacetyldiglycerides extracted from deer antlers were reported to stimulate the proliferation of HSCs and bone marrow stromal cells [56]. In addition, PLAG was demonstrated to increase the population of myeloid progenitor cells, which were decreased by 5-FU treatment in a murine model of chemotherapy-induced thrombocytopenia [57]. On the basis of the previous literature, the regulatory effect of PLAG on hematopoietic functions may contribute to the alleviation of 5-FU-induced hematological toxicity. In future studies, we will examine the effect of PLAG on the regeneration of hematopoietic stem cells after chemotherapy treatment.

Mice have genetic similarities with human, which confers mice as a useful animal model to study mechanisms and investigative therapeutics of human diseases [58,59]. However, mice have many hematological differences from humans including lower peripheral neutrophil and monocyte counts, a higher percentage of lymphocytes in blood and bone marrow, and a higher peripheral platelet count [60]. Furthermore, the chemotherapy treatment in cancer patients involves multiple cycles and is over a long period [61]. Thus, the patterns of hematological toxicity observed in a clinical setting would be different from a well-controlled nonclinical setting. Nevertheless, we found that 5-FU induces hematological toxicity not only in humans, but also in mice, but the above-mentioned differences should be kept in mind when translating our nonclinical results to human cases. Likewise, we expect that PLAG will exert its efficacy on the hematological toxicity in cancer patients with 5-FU-based chemotherapy regimen, but the optimal timing and dosing schedule should be investigated to enhance its efficacy in cancer patients.

## 4. Materials and Methods

### 4.1. Chemicals

PLAG and PLH were produced by and obtained from Enzychem Lifescience Corporation (Jecheon, Korea). 5-Fluorouracil and olive oil were purchased from Sigma Aldrich (St. Louis, MO, USA). 

### 4.2. Animals

Specific-pathogen-free male BALB/c mice (seven weeks of age) were obtained from Koatech Co. (Pyongtaek, Korea). Upon receipt, the mice were housed, five per cage, in a specific pathogen-free facility, and acclimatized for one week under conditions of consistent temperature and normal light cycles. All the animals were fed a standard mouse diet with water allowed ad libitum. All experimental procedures were approved by the Institutional Animal Care and Use Committee of the Korea Research Institute of Bioscience and Biotechnology (the ethic code is KRIBB-AEC-18197; approval date: 21 August 2018) and were performed in compliance with the Guide for the Care and Use of Laboratory Animals by the National Research Council and Korean National Laws for Animal Welfare.

### 4.3. Establishment of a Mouse Model of 5-FU-Induced Hematological Toxicity

Male BALB/c mice (eight weeks of age, five mice per group) were randomly divided into five groups—the control group; and the PLAG 100, 200, and 400 mg/kg-treated groups. The mice were intraperitoneally (i.p.) injected once with 100 mg/kg of 5-FU to induce hematologic toxicity. PLAG was suspended in sterile phosphate buffered saline (PBS) and was orally administrated beginning on the same day of 5-FU injection, continuing daily until day 15. The control group was administered sterile PBS only during the experiment. For the comparative experiment, 200 mg/kg of olive oil (Sigma-Aldrich, St. Louis, MO, USA) was re-suspended in sterile PBS. Mice were administered with PLAG, olive oil, or sterile PBS beginning on the same day of 5-FU injection and continuing daily until day 30. The whole blood was collected from the orbital sinuses using EDTA-free capillary tubes (Kimble Chase Life Science and Research Products LLC, Rockwood, FL, USA) and collection tubes containing K3EDTA (Greiner Bio-One International, Kremsmünster, Austria). The blood cells were counted and classified by complete blood count (CBC) analysis using Mindray BC-5000 auto-hematology analyzer (Shenzhen Mindray Biomedical Electronics, Guangdong Sheng, China). After CBC analysis, plasma was separated from the whole blood by centrifugation and stored at −80 °C for further analysis.

### 4.4. Measurement of Potential Biomarkers for 5-FU-Induced Hematological Toxicity 

The concentration of murine CXCL1, CXCL2, and IL-6 in mouse plasma was measured using a mouse premixed multi-analyte kit (R&D systems, Minneapolis, MN, USA) according to the manufacturer’s instructions. Fluorescence intensities were measured using a MAGPIX multiplexing system (Luminex Corporation, Austin, TX, USA). The concentration of murine C-reactive protein (CRP) in mouse plasma was measured by the enzyme-linked immunosorbent assay (ELISA) using CRP ELISA kit (R&D systems, Minneapolis, MN, USA) according to the manufacturer’s instructions. Optical densities were measured at 450 nm using a Bio-Rad Model 550 microplate reader (Bio-Rad Laboratories, Irvine, CA, USA). The concentration of analytes in plasma was calculated from standard curves generated by a curve-fitting program.

### 4.5. Statistical Analyses 

The results were expressed as the mean ± standard deviation (SD). Statistical analysis was performed using one-way analysis of variance (ANOVA) followed by Turkey–Kramer post-test using GraphPad Prism version 8.0 (GraphPad Software, San Diego, CA, USA), and *p* values < 0.05 were considered statistically significant. Mann–Whitney test was used to compare duration and time to recovery from neutropenia, monocytopenia, and thrombocytopenia between the control and PLAG or olive oil-treated cohorts. The correlation between two continuous variables was determined by Pearson correlation coefficient.

## 5. Conclusions

Our study shows that the administration of PLAG significantly mitigated 5-FU-induced hematological toxicitiy including neutropenia, monocytopenia, thrombocytopenia, and thrombocytosis. Moreover, it remarkably decreased plasma levels of pro-inflammatory cytokine/chemokines, which increased consistently with the occurrence time of neutropenia, monocytopenia, and thrombocytopenia. Together, we show that PLAG has therapeutic potential as a mitigator for the improvement of 5-FU-induced hematological toxicity.

## Figures and Tables

**Table 1:** The mean first day and mean duration of neutropenia (absolute neutrophil count (ANC) < 500 cells/µL), monocytopenia (peripheral monocyte counts < 150 cells/µL), and thrombocytopenia (>50% reduction from baseline) in control and 1-palmitoyl-2-linoleoyl-3-acetyl-rac-glycerol (PLAG)-treated mice injected with 5-Fluorouracil (5-FU) (100 mg/kg).

Treatment	Mean First Day of Neutropenia (±SE, Range)	Mean Duration of Neutropenia in Days (±SE, Range)	Mean First Day of Monocytopenia (±SE, Range)	Mean Duration of Monocytopenia in Days (±SE, Range)	Mean First Day of Thrombocytopenia (±SE, Range)	Mean Duration of Thrombocytopenia in Days (±SE, Range)
Control	4.7 ± 0.2 (3–5)	6.0 ± 0.4 (5–7)	3.7 ± 0.4 (3–5)	7.5 ± 0.6 (5–9)	4.2 ± 0.4 (3–5)	2.1 ± 0.3 (1–3)
PLAG 50	5.1 ± 0.1 (5–6)	5.3 ± 0.3 (4–7)	5.1 ± 0.1 (5–6)	5.6 ± 0.4 (4–7)	3.7 ± 0.7 (3–5)	1.7 ± 0.3 (1–2)
PLAG 100	5.0 ± 0.0 (5–5)	4.6 ± 0.4 (2–5)	4.2 ± 0.4 (3–5)	4.5 ± 0.8 (2–7)	3.0 ± 0.0 (3–3)	2.0 ± 0.0 (2–2)
PLAG 200	5.0 ± 0.0 (5–5)	3.1 ± 0.7 (1–5)	5.8 ± 0.2 (5–6)	1.2 ± 0.2 (1–2)	3.0 ± 0.0 (3–3)	2.0 ± 0.0 (2–2)
PLAG 400	4.7 ± 0.0 (3–5)	2.9 ± 0.7 (1–5)	6.0 ± 0.0 (6–6)	1.0 ± 0.0 (1–1)	3.0 ± 0.0 (3–3)	2.0 ± 0.0 (2–2)
Two-sided *p* values (control vs. PLAG 50)	0.5333	0.2168	0.0326 *	0.0089 **	0.5455	0.6667
Two-sided *p* values (control vs. PLAG 100)	>0.9999	0.0513	0.0131 *	0.0006 ***	0.0808	0.7394
Two-sided *p* values (control vs. PLAG 200)	>0.9999	0.0109 *	0.0002 ***	0.0003 ***	N/A	N/A
Two-sided *p* values (control vs. PLAG 400)	1	0.0054 **	0.0002 ***	0.0003 ***	N/A	N/A

* indicates statistical significance between control and PLAG-treated groups. * *p* < 0.05, ** *p* < 0.01, *** *p* < 0.001.

**Table 2:** Number of mice of severe neutropenia (ANC < 100 cells/µL), and mean first day and mean duration of severe neutropenia in control and PLAG-treated mice injected with 5-FU (100 mg/kg).

Treatment	Number of Mice of Severe Neutropenia	Mean First Day Severe Neutropenia (±SE, Range)	Mean Duration of Severe Neutropenia (±SE, Range)
Control	8/8	5.0 ± 0.0 (5–5)	5.1 ± 0.5 (2–7)
PLAG 50	8/8	5.4 ± 0.2 (5–6)	3.2 ± 0.6 (1–5)
PLAG 100	8/8	5.4 ± 0.2 (5–6)	2.1 ± 0.6 (1–5)
PLAG 200	0/8	None	N/A
PLAG 400	0/8	None	N/A
Two-sided *p* values (Control vs. PLAG 50)	N/A	0.2	0.0177
Two-sided *p* values (Control vs. PLAG 100)	N/A	0.2	0.0079
Two-sided *p* values (Control vs. PLAG 200)	N/A	N/A	N/A
Two-sided *p* values (Control vs. PLAG 400)	N/A	N/A	N/A

**Table 3:** Mean nadir and mean number of days to recovery of ANC, peripheral monocytes, and platelets in control and PLAG-treated mice injected with 5-FU (100 mg/kg).

Treatment	Nadir of ANC (cells/μL)	Mean Number of Days to Recovery—ANC ≥ 500/μL (± SE, Range)	Nadir of Monocytes (MON) (cells/μL)	Mean Number of Days to Recovery—MON ≥ 150/μL (±SE, Range)	Nadir of Platelets (PLT) (cells/nL)	Mean Number of Days to Recovery—PLT ≥ 1000 × 10^3^/μL (±SE, Range)
Control	3.2 ± 1.4	11.0 ± 0.4 (10–12)	2.1 ± 1.1	11.2 ± 0.4 (10–12)	409.6 ± 12.2	8.5 ± 0.6 (7–10)
PLAG 50	17.6 ± 5.2	10.5 ± 0.3 (10–12)	16.4 ± 5.6	10.7 ± 0.4 (10–12)	689.5 ± 28.5	5.7 ± 0.2 (5–7)
PLAG 100	27.5 ± 6.7	9.6 ± 0.4 (7–10)	32.5 ± 4.1	8.9 ± 0.5 (7–10)	660.6 ± 19.8	6.4 ± 0.6 (5–10)
PLAG 200	236.2 ± 36.4	8.1 ± 0.7 (6–10)	106.2 ± 22.6	7.0 ± 0.0 (7–7)	786.1 ± 37.7	4.9 ± 0.3 (3–6)
PLAG 400	245 ± 19.8	8.1 ± 0.7 (6–10)	142.5 ± 26.2	7.0 ± 0.0 (7–7)	813.1 ± 48.7	5.4 ± 0.2 (5–6)
Two-sided *p* values (control vs. PLAG 50)	0.0123 *	0.6084	0.0076 **	0.2104	0.0002 ***	0.0008 ***
Two-sided *p* values (control vs. PLAG 100)	0.0016 **	0.0513	0.0002 ***	0.0011 *	0.0002 ***	0.0194 *
Two-sided *p* values (control vs. PLAG 200)	0.0002 ***	0.0109 *	0.0002 ***	0.0009 ***	0.0002 ***	0.0002 ***
Two-sided *p* values (control vs. PLAG 400)	0.0002 ***	0.0109 *	0.0002 ***	0.0007 ***	0.0002 ***	0.0002 ***

* indicates statistical significance between control and PLAG-treated groups. * *p* < 0.05, ** *p* < 0.01, *** *p* < 0.001.

**Table 4:** Effect of PLAG, olive oil, and palmitic linoleic hydroxyl glycerol (PLH) administration in 5-FU-induced hematological toxicity.

Title	Treatment				Two-Sided *p* Values (Control vs.)		
	Control	PLAG	Olive Oil	PLH	PLAG	Olive Oil	PLH
Mean First Day of Neutropenia	5.0 ± 0.0 (5–5)	5.0 ± 0.0 (5–5)	5.0 ± 0.0 (5–5)	5.0 ± 0.0 (5–5)	1	1	1
Mean Duration of Neutropenia	5.0 ± 0.0 (5–5)	2.0 ± 0.0 (2–2)	8.0 ± 1.2 (5–10)	7.0 ± 0.0 (7–7)	0.06	0.0269 *	0.1824
Number of mice of Severe Neutropenia	5/5	0/5	5/5	5/5	N/A	N/A	N/A
Mean First Day of Severe Neutropenia	5.0 ± 0.0 (5–5)	N/A	5.0 ± 0.0 (5–5)	5.0 ± 0.0 (5–5)	N/A	1	1
Mean Duration of Severe Neutropenia	5.0 ± 0.0 (5–5)	N/A	5.0 ± 0.0 (5–5)	5.0 ± 0.0 (5–5)	N/A	1	1
Nadir of ANC (cells/μL)	28.0 ± 7.3	344.0 ± 90.8	18.0 ± 2.0	8.2 ± 1.8	0.0008 ***	0.9986	0.9896
Mean Number of Days to Recovery ANC ≥ 500 μL (±SE, range)	10.0 ± 0.0 (10–10)	7.0 ± 0.0 (7–7)	12.4 ± 1.2 (10–15)	12.0 ± 0.0 (12–12)	0.0375 *	0.0577	0.132
Mean First Day of Monocytopenia	4.2 ± 0.5 (3–5)	6.0 ± 1.0 (5–7)	4.2 ± 0.5 (3–5)	3.8 ± 0.8 (1–5)	0.4274	1	0.9661
Mean Duration of Monocytopenia	6.2 ± 0.8 (5–9)	2.5 ± 0.5 (2–3)	7.6 ± 1.7 (5–12)	6.2 ± 0.5 (5–7)	0.1327	0.6458	>0.9999
Nadir of MON	20.2 ± 8.2	198.0 ± 37.5	20.0 ± 3.2	16.0 ± 2.5	<0.0001 ***	>0.9999	0.9986
Mean Number of Days to Recovery MON ≥ 150/μL (±SE, range)	10.4 ± 0.4 (10–12)	8.5 ± 1.5 (7–10)	10.8 ± 0.5 (10–12)	10.8 ± 0.5 (10–12)	0.2488	0.9454	0.9454
Mean First Day of Thrombocytopenia	4.6 ± 0.4 (3–5)	3.0 ± 0.0 (3–3)	4.6 ± 0.4 (3–5)	4.6 ± 0.4 (3–5)	0.3979	>0.9999	>0.9999
Mean Duration of Thrombocytopenia	2.4 ± 0.4 (2–4)	2.0 ± 0.0 (2–2)	2.4 ± 0.4 (2–4)	2.4 ± 0.4 (2–4)	0.976	>0.9999	>0.9999
Nadir of PLT	509.0 ± 13.2	868.8 ± 70.2	518.8 ± 50.3	462.8 ± 17.8	0.0002 ***	0.9986	0.8826
Mean Number of Days to Recovery PLT ≥ 1000 × 10^3^/μL (±SE, range)	8.8 ± 0.7 (7–10)	5.0 ± 0.0 (5–5)	7.6 ± 0.6 (7–10)	8.2 ± 0.7 (7–10)	0.0048 **	0.5361	0.9013

* *p* < 0.05, ** *p* < 0.01, *** *p* < 0.001.
